# Decoding the role of platelets in tumour metastasis: enigmatic accomplices and intricate targets for anticancer treatments

**DOI:** 10.3389/fimmu.2023.1256129

**Published:** 2023-12-01

**Authors:** Jessie Zhao, Angela Huang, Johannes Zeller, Karlheinz Peter, James D. McFadyen

**Affiliations:** ^1^ Department of Clinical Haematology, Alfred Hospital, Melbourne, VI, Australia; ^2^ Australian Centre for Blood Diseases, Monash University, Melbourne, VI, Australia; ^3^ Atherothrombosis and Vascular Biology Laboratory, Baker Heart and Diabetes Institute, Melbourne, VI, Australia; ^4^ Department of Plastic and Hand Surgery, Medical Center – University of Freiburg, Medical Faculty of the University of Freiburg, Freiburg, Germany; ^5^ Department of Cardiology, Alfred Hospital, Melbourne, VI, Australia; ^6^ Department of Cardiometabolic Health, The University of Melbourne, Parkville, VI, Australia; ^7^ Department of Medicine, Monash University, Melbourne, VI, Australia

**Keywords:** cancer, platelet, tumour microenvirnonment, anti platelet therapy, metastasis (cancer metastasis)

## Abstract

The canonical role of platelets as central players in cardiovascular disease by way of their fundamental role in mediating thrombosis and haemostasis is well appreciated. However, there is now a large body of experimental evidence demonstrating that platelets are also pivotal in various physiological and pathophysiological processes other than maintaining haemostasis. Foremost amongst these is the emerging data highlighting the key role of platelets in driving cancer growth, metastasis and modulating the tumour microenvironment. As such, there is significant interest in targeting platelets therapeutically for the treatment of cancer. Therefore, the purpose of this review is to provide an overview of how platelets contribute to the cancer landscape and why platelets present as valuable targets for the development of novel cancer diagnosis tools and therapeutics.

## Introduction

The relationship between platelets, thrombosis, and cancer has long been acknowledged. Already in the nineteenth century, Armand Trousseau reported the association between cancer and thrombosis, describing migratory thrombophlebitis as a presenting feature of visceral cancer (1). Thrombocytosis is a frequent finding in cancer patients, and the link between thrombocytosis and malignancy was first noted by Reiss et al. in 1872 (2). Thrombocytosis remains widely recognised as an adverse prognostic marker correlating with shortened survival in a wide range of common malignancies, including lung, breast, colorectal, gastric, renal, and ovarian cancers (3–11).

There is now a large and constantly growing body of evidence illustrating the bidirectional relationship between platelets and cancer. In 1968, Gasic et al. first described the correlation between platelets and metastatic potential, demonstrating that neuraminidase-induced thrombocytopenia was able to inhibit tumour metastasis in mice and that this antimetastatic effect could be reversed by the transfusion of platelet-rich plasma (12). Further experimental evidence identified that thrombopoietic cytokine production by tumour cells, specifically interleukin (IL)-6, induces platelet production, thereby creating a feedback loop that further enhances tumour growth (10). Here, platelet-derived factors not only offer stability to the tumour vasculature but have the capacity to increase the metastatic potential of tumour cells (13, 14).

Notably, platelets have been shown to influence the efficacy of cancer therapies. Large cohort studies have revealed that in comparison to the general population, the risk of developing thrombosis is much higher in cancer patients (15). Elevated platelet count in ovarian cancer patients is associated with higher relapse rates and lower response rates to taxane chemotherapy, indicating that platelets may offer cancer cells protection against apoptosis (16). Experimental models of adenocarcinoma have also demonstrated that the presence of platelets conferred increased tumour cell survival rates against treatment with standard anticancer drugs, 5-fluorouracil and paclitaxel (17). These findings emphasise the intimate and bidirectional crosstalk that exists between platelets and tumours. This review will discuss the mechanisms by which platelets and tumours interact and explore the consequences of platelet-tumour interactions. Finally, we will provide a critical appraisal of how these new insights may be exploited for the optimisation of cancer treatments.

## Platelet-tumour crosstalk

There has been a shift in the understanding of cancerous tumours in recent years, where tumours are no longer seen as mere insular masses of proliferating cancer cells. Indeed, there is now a widely accepted appreciation that recruited immune cells in the surrounding tumour environment play fundamental roles in mediating tumour growth and behaviour (18, 19). This dynamic crosstalk between cancer cells and non-malignant cells creates the tumour microenvironment (TME), and our growing awareness of the importance of the TME in all steps of tumorigenesis has led to an increased emphasis on therapies that may target elements of the TME (19, 20).

**Figure 1:**
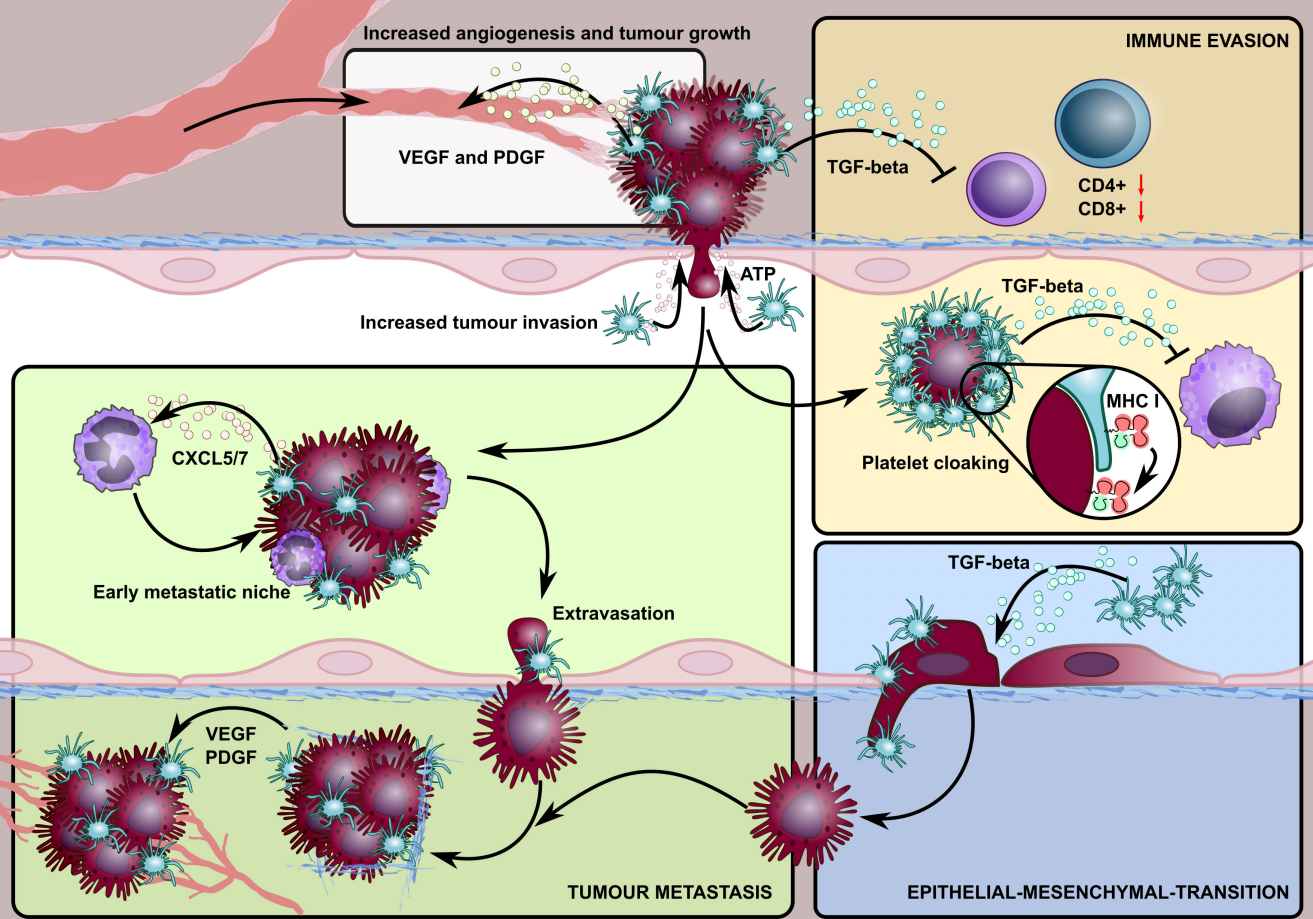
A schematic overview highlighting the involvement of platelets in different stages of the cancer lifecycle. Platelet granules contain growth factors, such as VEGF and PDGF that promote tumour angiogenesis. Platelets also stabilise neovessels and maintain tumour vascular integrity, in a process mediated by platelet focal adhesion kinase, where platelets within intra-tumoural blood vessels extravasate to the tumour microenvironment and enhance the survival rate of detached cancer cells. Activated platelets enable tumour cells to escape detection by the immune system (immune evasion) by 1) adhering to and ‘cloaking’ disseminating tumour cells, offering protection against NK cell detection by transferring their MHC class I molecules to tumour cells, and 2) releasing TGF-β which suppresses T cell immune responses in the tumour microenvironment. The release of platelet-derived TGF-β is also critical in promoting tumour epithelial-mesenchymal transformation which enhances their metastatic potential. Platelet-derived ATP activates endothelial cells, triggering an opening that allows for the endothelial transmigration of tumour cells. Lastly, the release of platelet-derived chemokines, CXCR5/7, recruits granulocytes to form platelet-tumour aggregates, allowing the formation of a specialised microenvironment, the so-called early metastatic niche, which promotes efficient metastatic seeding.

Beyond their canonical role in haemostasis and thrombosis, platelets are also critical immune modulators of the innate and adaptive immune responses, by way of their surface expression of adhesion receptors and capacity to release an array of cytokines and growth factors from intracellular granules (21–23). Several platelet adhesion receptors relevant to haemostasis have been directly implicated in malignancy, in this regard, platelets and tumour cells maintain a complex, bidirectional interaction directly linked to the survival of tumour cells (**Figure 1**) (24, 25).

Various studies have now demonstrated that tumour cells induce platelet activation in a process known as tumour cell-induced platelet aggregation (TCIPA). Interestingly, this facultative ability of tumour cells also correlates with their metastatic potential (26, 27). TCIPA confers several survival advantages to the platelet-coated tumour cells – namely, the evasion of immune detection, platelet release of growth factors supporting tumour growth and protection against the potentially detrimental effects of high shear forces of the flowing blood (26, 28–30). Moreover, TCIPA facilitates the embolization of large tumour-platelet clusters within the microvasculature at the new extravasation site (26, 28). Although detailed mechanisms of these interactions remain to be elucidated, tumour cells have demonstrated the ability to activate and aggregate platelets through thrombin generation, the release of ADP, thromboxane A2 (TXA2) and matrix metalloproteinase 2 (MM2) (31–33).

**Figure 2:**
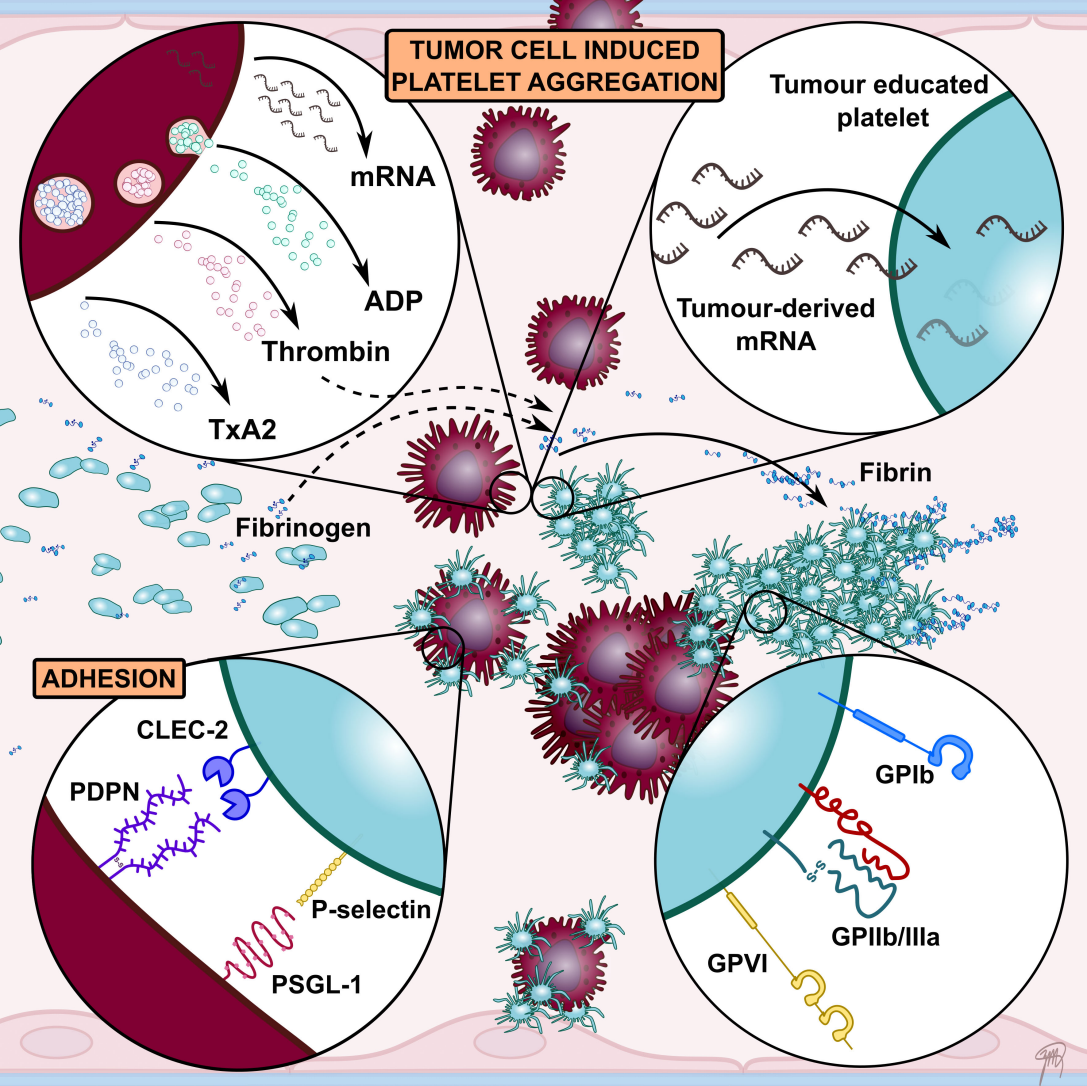
A schematic overview highlighting key interactions between platelets and tumour cells facilitating tumour cell-induced platelet aggregation (TCIPA). Tumour cells can activate and aggregate platelets directly through the release of platelet agonists such as ADP and thrombin. A number of platelet adhesion receptors have been implicated to contribute to TCIPA, including GPIIb/IIIa, GPIb, GPVI, P-selectin and CLEC-2. Their involvement in promoting tumour growth and metastasis has led to numerous preclinical studies investigating platelet receptors as therapeutic targets to enhance cancer therapy. Tumour cells have also been shown to alter platelet mRNA content. These so called tumour-educated platelets (TEPs) have been investigated as novel liquid biopsy tool for cancer diagnosis.

Major platelet receptors have been described to mediate platelet-tumour cell interactions, including P-selectin, glycoproteins (GP) IIb/IIIa, GPIb, GPVI, and C-type lectin-like receptor 2 (CLEC-2) (25, 34–37). As will be discussed below, the role of these platelet receptors in governing platelet-tumour interactions has led to significant interest in the therapeutic targeting of platelets for the treatment of cancer (**Figure 2**).

## Platelets support tumour growth

Angiogenesis, – the formation of new blood vessels – is fundamental for tumour growth and spread (38). Platelet α-granules are a major reservoir for pro-angiogenic factors including vascular endothelial growth factor (VEGF), platelet-derived growth factor (PDGF) and basic fibroblast growth factor (bFGF) (39). Therefore, as a consequence of the ability of tumour cells to induce platelet activation, platelets serve as transporters that release angiogenic regulators, thus enhancing tumour angiogenesis (40). In murine models, platelet depletion reduced the formation of bFGF-induced neoangiogenesis in the cornea (41). Moreover, in thrombocytopenic mice bearing the Lewis lung carcinoma and B16F10 melanoma, the occurrence of haemorrhage was markedly increased, implicating that platelets serve to stabilize newly formed vessels and maintain tumour vascular integrity (13, 41). The latter study also found that tumoral bleeding occurred immediately following antibody-induced platelet depletion, resulting in intra-tumour haemorrhage, reduced tumour cell proliferation and increased tumour necrosis (13). Although the transfusion of platelets was able to arrest such bleeding, the transfusion of thrombin-activated degranulated platelets could not, highlighting the notion that granular contents in platelets are crucial for the regulation and formation of tumour vasculature (13). Moreover, preclinical studies have demonstrated that platelet inhibition with aspirin is associated with a reduction of platelet-mediated cancer cell proliferation and metastasis (42). These effects were due to the inhibition of platelet COX-1 and the subsequent suppression of thromboxane A2 and prostaglandin E2 generation, thereby limiting metastasis in a colon cancer model (42). Additionally, platelet inhibition with aspirin diminishes platelet VEGF release, which leads to a suppression of angiogenesis in breast cancer cell lines (43).

Importantly, platelets in cancer patients are functionally altered. Compared to healthy controls, platelets from early breast cancer patients were found to release an increased amount of VEGF upon activation with thrombin and tissue factor (44). Similar clinical observations were also made where platelet lysates from breast cancer patients and tumour aspirates from sarcoma patients contained higher concentrations of VEGF compared to healthy controls (45, 46).

More recently, it has emerged that platelets within intra-tumour blood vessels can extravasate into the extracellular matrix of the TME in a process mediated by platelet focal adhesion kinase (FAK) (47). In a mouse model of platelet-specific FAK deficiency, platelet infiltration into the TME was reduced (47). Additionally, the co-incubation of platelets with human ovarian cancer cells reduced the rate of anoikis (detachment-induced apoptosis), leading to the increased survival of detached cancer cells and enhanced metastatic potential (48). Following platelet co-incubation, signalling pathway analysis revealed that yes-associated protein 1 (YAP1), a transcription co-regulator, was differentially regulated (48). The phosphorylation of YAP1 was observed to be associated with platelet-mediated protection from anoikis, implicating that the activation of YAP1 in the signalling pathway results in the expression of a pro-survival gene signature, promoting tumour survival and metastasis (48).

## Platelets support tumour invasion and metastasis

Cancer metastasis is a cascade of events involving the detachment of metastatic cells from the primary tumour, extravasation into the bloodstream, evasion of immune detection, and finally the settlement and growth at distant organs (49, 50). Sequalae related to metastasis remains the leading cause of cancer mortality, accounting for 90% of all cancer-related deaths (49). Recent data have highlighted several critical roles platelets play in regulating these key steps required for tumour metastasis, which is underscored by the observation that platelet inhibition or depletion affords protection from metastasis in a range of experimental tumour models (12, 51, 52).

During the haematogenous phase of metastasis, tumour cells are in a hostile environment interacting directly with effectors of the host immune system, a stage where the majority of circulating tumour cells do not survive (53). Natural killer (NK) cells play an important role in the immunosurveillance of tumours by eliminating malignant cells through direct cytolysis or interferon (IFN)-γ secretion to induce an efficient T-cell-mediated response, preventing both local tumour progression and metastasis (54). However, platelets activated by metastatic tumours in the bloodstream assist with tumour dissemination by adhering to and ‘cloaking’ circulating tumour cells, masking them from NK cell recognition and subsequent destruction (14). Here, platelets transfer their major histocompatibility complex (MHC) class I molecules to metastasising tumour cells, imparting a platelet-derived “pseudo-self” phenotype to tumour cells thereby hiding malignant cells from NK cell recognition (55). Additionally, activated platelets can inhibit NK cell effector functions through the release of humoral factors such as TGF-β, which downregulates NKG2D – an activation receptor of NK cells to sense stress-associated molecules, in which its depletion results in the inhibition of NK cytotoxicity and IFN-γ transcription (56, 57). In doing so, platelets undermine the second mainstay of NK cells to recognise stress-induced ligands, further impairing NK cell immunosurveillance (57).

## Platelet adhesion receptors mediating tumour growth and metastasis

### Glycoprotein IIb/IIIa

GPIIb/IIIa is the most abundant platelet surface receptor and has been demonstrated to play an important role in cancer growth and metastasis. Indeed, pharmacological inhibition of GPIIb/IIIa with eptifibatide reduced cell migration and invasion of breast cancer carcinoma cells *in vitro* (34). Furthermore, the targeted deletion of β3 integrin has been demonstrated to afford protection against osteolytic bone metastasis in a mouse model of melanoma (52). Accordingly, conformation-specific antibodies, using a single-chain variable fragment (scFv) antibody platform, targeting the activated conformation of GPIIb/IIIa on platelets has been used as a molecular targeting epitope for imaging tumours in several types of cancers in mice (58). Subsequent studies from the same group also explored the potential of an scFv antibody-drug conjugate that targeted the activated GPIIb/IIIa receptor whilst carrying a potent chemotherapeutic microtubule inhibitor (59). This antibody-drug conjugate demonstrated efficacy in a murine xenograft model of triple-negative breast cancer, highlighting its application to target platelets in the setting where tumour cell-specific epitopes are absent (59).

### Glycoprotein Ibα

Platelet GPIbα is a subunit of the GPIb-IX-V receptor complex constitutively and exclusively expressed on the plasma membrane of circulating platelets and megakaryocytes (60). However, the precise role of GPIbα in mediating tumour metastasis remains controversial. Indeed, previous studies have been conflicting with pulmonary metastasis being reduced in GPIbα-deficient mice yet antibody-mediated functional inhibition of GPIbα led to a significant increase in pulmonary metastasis (35, 61). Similarly, the deletion of von Willebrand factor (vWF), the major ligand of GPIbα, markedly increased pulmonary metastasis following the injection of tumour cells (62). It is hypothesized that the adverse metastatic effects observed with GPIbα blockage are a result of abolishing interactions between GPIbα and vWF, which occurs physiologically in high shear stress conditions (35). Instead, tumour cells are arrested in mechanically less stressful environments, favouring their survival and growth (35, 60). Furthermore, some anti-GPIbα monoclonal antibodies have been shown to induce platelet activation, suggesting the possibility of enhancing TCIPA through platelet aggregation, resulting in the observed increase in metastasis in experimental models (21, 63).

### Glycoprotein VI

GPVI is a platelet-specific receptor with a critical role in mediating collagen-induced platelet activation. GPVI deficiency led to a 50% decrease in metastasis in B16F10.1 melanoma and Lewis lung carcinoma cells (36). Moreover, GPVI has been shown to support platelet adhesion to breast and colon cancer cells through binding to galectin-3, which resulted in the promotion of tumour extravasation (64). Recently, the inhibition of GPVI was shown to increase intra-tumour haemorrhage, which was associated with a decrease in tumour growth and enhanced efficacy of chemotherapy (65). Further supporting this finding, the repurposing of revacept, a soluble dimeric GPVI-Fc fusion protein developed for the treatment of atherothrombotic diseases, has been demonstrated in a model of colon carcinoma to inhibit aberrant COX-2 expression and tumour EMT (66). Whilst these data support the important role of GPVI in cancer growth and metastasis, further investigation of GPVI inhibition is required. This is particularly relevant since the reported intra-tumoral haemorrhage associated with GPVI inhibition may in turn limit the feasibility of clinical translation given the potential to develop deleterious sequelae of bleeding in metastatic lesions, particularly intracerebral lesions.

### P-selectin

P-selectin belongs to the selectin family of cell adhesion molecules. In platelets, P-selectin is stored in the α-granules and is rapidly translocated to the platelet surface via exocytosis upon cell activation (67). P-selectin has been demonstrated to bind to a variety of human cancers and cancer-derived cell lines *in vitro*, including colon cancer, small cell lung cancer, breast cancer, melanoma, neuroblastoma and adenoid cancer, where P-selectin has been shown to contribute to tumour cell extravasation (68–73). Accordingly, several studies have shown that P-selectin-deficient mice demonstrate attenuation of metastasis (50, 74, 75). Furthermore, the use of the anticoagulant heparin – a highly sulphated glycosaminoglycan that binds and inhibits P-selectin – was able to attenuate metastasis in mouse models (50, 75, 76). However, the benefit of low molecular weight heparin on the survival of cancer patients in large, randomised clinical trials has not been replicated (77, 78).

### CLEC-2

CLEC-2 is a platelet activation receptor expressed almost exclusively in platelets and megakaryocytes and at very low levels in liver Kupffer and sinusoidal cells (79). Originally identified as a platelet-activating receptor to snake venom rhodocytin, CLEC-2 induces platelet activation via similar signalling pathways to that of GPVI, resulting in platelet aggregation (80). The only known endogenous ligand of CLEC-2, podoplanin, has been identified on the surface cells from osteosarcoma, squamous cell carcinoma and glioblastoma tumours (81–84). The surface expression of podoplanin on tumour cells induces platelet binding, and a study examining 26 cell lines of the mouse colon adenocarcinoma observed that metastatic clones were associated with higher podoplanin expression and a greater platelet aggregation capacity (85). Additionally, in experimental models of lung metastasis, the use of an anti-podoplanin antibody was able to inhibit platelet aggregation and markedly reduced the number of sites of metastatic foci (37).

### Platelets promote tumour cell epithelial-mesenchymal transition

Recent data have highlighted a key step governing the metastatic potential of tumour cells is the ability of primary tumour cells to undergo epithelial-mesenchymal transition (EMT)-like transformation, which promotes tumour cell motility, invasion, and dissemination (86). This process is enhanced by TGF-β, as evidenced in metastatic breast cancer models, where an induced increase of TGF-β levels is associated with increased circulating tumour cells and metastasis in the lung (87). Such findings were also reflected in the clinical setting where increased plasma levels of TGF-β correlated with colorectal cancer progression, and an increase in immunostaining of TGF-β has been implicated with metastasis in prostate, breast and colon cancer patients (88–91). Importantly, platelets are a major source of bioavailable TGF-β, and seminal work from the Hynes group demonstrated that the metastatic capacity of tumour cells can be further increased by active signals from platelets in which they come into transient contact during their transit in the blood (86, 92). This notion is supported by the finding that pre-treating tumour cells with platelets lacking TGF-β were not able to enhance metastasis formation (86). Further investigations revealed that platelet-derived TGF-β appears to be critical in inducing a pro-metastatic gene profile in tumour cells, strongly upregulating EMT-related genes through activation of the Smad signalling pathway (86). Interestingly, platelet-derived TGF-β alone is insufficient to support metastasis *in vivo*, as tumours treated with platelets but not releasates from activated platelets were able to undergo EMT (86). Rather, tumour cell NF-κB signalling through direct platelet-tumour cell contact is required in synergy with platelet-derived TGF-β-Smad activation to prime tumour cells for metastasis (86).

Critically, platelets facilitate the early phases of metastatic seeding. Activated platelets release ATP, activating endothelial cells via the P2Y purinoreceptor 2 (P2Y2) receptor, which triggers an opening in the endothelial barrier for endothelial transmigration of tumour cells to occur (93). Importantly, mice deficient in P2Y2 or lacking platelet-derived ATP demonstrated a marked reduction in metastasis (93). Moreover, platelets interact with tumour cells to help guide the formation of the early metastatic niche, a specialised microenvironment to promote metastatic progression (51). Upon entry into the circulation, tumour cells will quickly encounter platelets and granulocytes, and the release of platelet-derived chemokines CXCR5/7 from tumour-bound platelets actively recruits granulocytes to platelet-tumour aggregates (51). This process appears essential for efficient metastatic seeding since platelet depletion or the inhibition of platelet chemokine signalling affords protection from metastasis (51).

These findings have been emphasised in ovarian cancer models, where the P2Y_12_ inhibitor, ticagrelor, as well as P2Y_12_ deficiency, were both associated with a reduction in tumour growth (94). Furthermore, ticlopidine, an irreversible P2Y_12_ receptor inhibitor, demonstrated inhibitory effects in the metastasis of rodent melanoma, Lewis lung carcinoma and hepatoma (95). Likewise, pulmonary metastasis was also inhibited through P2Y_12_ deficiency, decreasing the secretion of TGF-β from activated platelets, and diminished EMT in a Lewis lung carcinoma model (96). Moreover, the use of P2Y_12_ inhibitors as adjunct therapies has been explored with data demonstrating that ticagrelor enhances the chemotherapeutic efficiency in pancreatic ductal adenocarcinoma, displaying synergism when combined with gemcitabine *in vitro* (97). The synergistic effect of P2Y_12_ receptor inhibitors has also been shown with cisplatin in 4T1 breast cancer mouse models (98).

### Platelets as modulators of the immune response in the TME

Recent clinical advancement in cancer therapy has been the adoption of immunotherapy as a means to harness the immune system for cancer treatment. However, not all patients respond to immunotherapy, which at least in part, likely reflects the differences in the immune constitution of the TME between individuals (99, 100). Recent findings have demonstrated that platelets play a key role in regulating T cell responses within the TME, potentially limiting the efficacy of immunotherapy (101). Platelets within the TME are capable of suppressing functional responses of CD4^+^ and CD8^+^ T cells through the release of TGF-β (101). Whilst platelets contribute significantly to the circulating pool of TGF-β, they also constitutively express the glycoprotein-A repetitions predominant (GARP) receptor – a cell surface docking receptor crucial for regulating the activation of latent TGF-β (102). It appears in this context, the ability of platelet GARP to capture and activate latent TGF-β systemically is the major contributor to the platelet immunosuppressive effects within the TME (101). More recent studies have demonstrated that thrombin is responsible for the cleavage of GARP on the surface of platelets to liberate biologically active TGF-β, presenting as a potential mechanism of cancer immune evasion (103). In support of this hypothesis, platelet depletion, anti-platelet therapy and direct thrombin inhibitors demonstrate efficacy in enhancing the anti-tumour effects of adoptive T-cell transfer therapy (ACT) and immunotherapy in preclinical cancer models (103).

## Exploiting the platelet cancer interplay for therapeutic benefit

### Tumour-educated platelets for cancer diagnosis

Platelets can in turn be altered or ‘educated’ by interactions with tumour cells, leading to platelets with a modified protein and RNA profile or a “tumour-educated platelet” (TEP) (104, 105). Tumour-mediated platelet education, either via direct or indirect mechanisms, has the potential to be exploited as a biomarker for liquid biopsy diagnostics.

Despite being anucleate cells, protein synthesis by platelets was described over half a century ago (106, 107). Since then, studies have revealed that platelets contain different types of RNA including protein-coding messenger RNAs (mRNAs) as well as a functional megakaryocyte-derived spliceosome for the processing of pre-mRNAs (108, 109). Activated platelets synthesise various functional proteins under signal-dependent controls, including the oncogene B-cell lymphoma-3, interleukin-1β (IL-1β), and tissue factor (110–112). The platelet transcriptome can be altered in response to external stimuli, including tumour cells via direct or indirect stimulation (105).

Platelets can be educated directly through the sequestration of tumour-derived microvesicles. Nilsson et al. established that cancer cells transfer mutant RNA into platelets in both *in vitro* and *in vivo* studies and showed that platelets from glioma and prostate cancer patients contained tumour-derived mRNA biomarkers EGFRvIII and PCA3, respectively (113). Furthermore, the fusion oncogene EML4-ALK – an essential growth driver in human cancer – was detected in TEPs from patients with non-small-cell lung cancer (NSCLC) and was proven relevant for prognosis and monitoring of therapy, whilst KLK3, FOLH1, and NPY genes were sequestered in platelets from patients with prostate cancer and enabled prediction of treatment outcomes (114, 115).

Platelets can also be indirectly altered through alternative RNA splicing, with various studies revealing that cancer patients display distinct platelet mRNA profiles. Calverley et al. reported that mRNA platelet profiles of metastatic NSCLC patients differ from control, where 197 out of the 200 platelet genes with the most altered expression were downregulated (116). Likewise, Nilsson et al. demonstrated a distinct RNA signature in TEPs from glioma patients, and Best et al. further characterised TEP RNA profiles and explored their diagnostic potential (104, 113). RNA sequencing combined with self-learning algorithms distinguished patients with localised and metastatic cancer from healthy controls with 96% accuracy, located the primary tumour for six different tumour types (NSCLC, colorectal cancer, glioblastoma, pancreatic cancer, hepatobiliary cancer, and breast cancer) with 71% accuracy, and allowed discrimination of tumour molecular subtypes (104). Later, Best et al. accurately used TEP RNA profiles in the detection of early- and late-stage NSCLC, further demonstrating that differential splicing was independent of age and various inflammatory conditions (117). The role of TEPs in haematological malignancies is less well defined but has been supported by a report of upregulated IL-1β detected by RNA sequencing of platelets in smouldering and plasma cell myeloma (118).

However, the exact mechanism of tumour cell-induced alternative RNA splicing in TEPs remains elusive. It is hypothesized that signal-dependent splicing of platelet RNA, differential binding of RNA-binding protein to the untranslated region of platelet RNA, differential transfer of RNA molecules into platelets by tumour-educated megakaryocytes, and a shift in the evolution of different platelet subpopulations towards RNA-rich reticulated platelets contribute to this process (109, 112, 117, 119–121). Whether the tumour-derived RNA or spliced RNA in TEPs is subsequently translated into functional proteins remains to be determined.

Liquid biopsies offer a simple, non-invasive, and repeatable alternative to tissue biopsies, allowing diagnosis, prognostication, individualised treatment, and treatment monitoring of cancer. Circulating tumour DNA or RNA and circulating tumour cells have been well studied, and given their abundance in peripheral blood, TEP-derived RNA and proteins have been proposed as novel cancer biomarkers in several cancers such as glioblastoma and lung, breast, colorectal, prostate, and ovarian cancers (104, 114, 115, 117, 122, 123). Several analysis methods have been reported, including next-generation sequencing with machine learning algorithms, quantitative reverse-transcriptase polymerase chain reaction, and proteomic analysis (104, 113, 117, 122, 124). However, technical standardisation and clinical validation are required before TEPs can be deployed in clinical practice as novel liquid biopsy biomarkers in cancer.

### Targeting platelets for cancer therapy

With an expanding body of evidence demonstrating the fundamental importance of platelets for tumour growth and survival, the prospect of targeting platelets for the treatment of cancer has gained significant interest. To date, as discussed above, a number of preclinical studies have focused on established anti-platelet drugs and antagonists that target platelet receptors and signalling pathways important for platelet-tumour interactions. However, data from clinical studies, regarding the effects on cancer remains more limited.

### Aspirin

Several clinical studies have investigated the use of aspirin as a chemopreventive agent. An early case-control study published in 1988 found aspirin use to be associated with a lower incidence of colorectal cancer (125). Various case-control and cohort studies followed, including a prospective mortality study of 662,424 patients that reported reduced death rates from colon cancer with regular aspirin use (126). Furthermore, the first two published randomised controlled trials in patients with previous colorectal cancer found a decreased risk of recurrence at one year with aspirin use (127, 128). The randomised controlled APACC trial similarly found daily aspirin decreased adenoma recurrence at one year, but this chemopreventive effect was not maintained at the final four-year colonoscopy although issues with methodology may have influenced the latter result (129, 130).

However, primary prevention trials have provided conflicting results. Trials initially designed to study cardiovascular disease prevention found reductions in colorectal cancer incidence with various doses of aspirin, although benefits were only observed after ten years (131, 132). Meta-analyses have demonstrated that aspirin modestly decreased the twenty-year risk of colon cancer incidence and mortality (133, 134). Despite these findings, the large randomised controlled ASPREE trial of healthy older adults found an increase in the secondary endpoint of overall mortality in patients randomised to low-dose aspirin, attributed predominantly to cancer-related deaths including from colorectal cancer (135). In a further analysis, aspirin use was associated with an increased risk of incident cancers that had metastasised or stage 4 cancers at diagnosis (136). These unanticipated findings may be due to the contrasting biological effects of aspirin in older adults and the lack of sufficient follow-up, findings from the observational trial ASPREE-XT evaluating the long-lasting effects of aspirin treatment in such demographics are awaited.

### P2Y_12_ inhibitors

Whilst preclinical studies have been encouraging, results from clinical studies for the use of P2Y_12_ inhibitors are mixed. Data from large randomised controlled trials in patients with cardiovascular disease have previously shown potential associations between P2Y_12_ receptor inhibitors and cancer. The TRITON-TIMI 38 trial found a higher incidence of cancer and cancer-related mortality in patients treated with prasugrel compared to clopidogrel as part of dual antiplatelet therapy, whilst the DAPT study and PEGASUS-TIMI trial reported higher incidences of cancer-related mortality with dual antiplatelet therapy using clopidogrel and ticagrelor, respectively (137–139). In contrast, a meta-analysis including 282,084 patients from six randomised controlled trials and three retrospective cohort studies found no increased risk of cancer incidence or mortality with P2Y_12_ receptor inhibitors (140).

### Perspectives and future directions

Our deepening understanding of platelets as important mediators of immunity and inflammation has afforded significant insights into their role in tumour growth and metastasis. However, a critical outstanding issue is how these findings can be translated to ultimately improve the therapeutic landscape and prognosis of patients with cancer. In this regard, the ongoing quest to develop novel anti-platelet approaches that do not cause bleeding may provide the ultimate hope to uncouple the potential benefit of antiplatelet therapy in cancer patients without the potentially significant bleeding risk. Whether specific aspects of platelet function that have been demonstrated to be important in cancer biology, such as platelet-derived ATP or TGF-β, can be inhibited without unwanted off-target effects remains to be tested. In the interim, further investigations regarding the potential of liquid biopsies for providing prognostic information and the role of anti-platelet therapies in modulating the tumour immune response are needed given the growing importance of immunotherapy in cancer therapy.

## Author contributions

JZh: Writing – original draft, Writing – review & editing. AH: Writing – original draft, Writing – review & editing. JZe: Writing – original draft, Writing – review & editing. KP: Writing – original draft, Writing – review & editing, Conceptualization. JM: Conceptualization, Writing – original draft, Writing – review & editing.

## Glossary

**Table:**

IL	Interleukin
TME	Tumour Microenvironment
TCIPA	Tumour Cell-Induced Platelet Aggregation
ADP	Adenosine Diphosphate
TXA2	Thromboxane A2
MM2	Matrix Metalloproteinase 2
GP	Glycoprotein
CLEC-2	C-Type Lectin-Like Receptor
VEGF	Vascular Endothelial Growth Factor
PDGF	Platelet-Derived Growth Factor
bFGF	Basic Fibroblast Growth Factor
FAK	Focal Adhesion Kinase
YAP1	Yes-Associated Protein 1
NK	Natural Killer
IFN	Interferon
MHC	Major Histocompatibility Complex
TGF	Tissue Growth Factor
EMT	Epithelial-Mesenchymal Transition
NF-κB	Nuclear Factor Kappa B
P2Y2	P2Y Purinoreceptor 2
ATP	Adenosine Triphosphate
CXCR	C-X-C Chemokine Receptor
CD	Clusters of Differentiation
GARP	Glycoprotein-A Repetitions Predominant
ACT	Adoptive T-cell Transfer Therapy
RNA	Ribonucleic Acid
mRNA	Messenger Ribonucleic Acid
TEP	Tumour-Educated Platelet
scFv	Single-Chain Variable Fragment
vWF	Von Willebrand Factor
PSGL	P-Selectin Glycoprotein Ligand
NSCLC	Non-Small-Cell Lung Cancer
EGRFvIII	Epidermal Growth Factor Receptor Variant Type II
PCA3	Prostate Cancer Antigen 3
EML4	Echinoderm Microtubule Associated Protein-Like 4
ALK	Anaplastic Lymphoma Kinase
KLK3	Kallikrein 3
FOLH1	Folate Hydrolase 1
NPY	Neuropeptide Y
COX	Cyclooxygenase
APACC	Prospective Study on Aspirin Efficacy in Reducing Colorectal Adenoma Recurrence
ASPREE	Aspirin in Reducing Events in the Elderly
TRITON-TIMI	Trial to Assess Improvement in Therapeutic Outcomes by Optimizing Platelet Inhibition With Prasugrel-Thrombolysis In Myocardial Infarction
DAPT	Dual Antiplatelet Therapy
PEGASUS-TIMI	Prevention of Cardiovascular Events in Patients With Prior Heart Attack Using Ticagrelor Compared to Placebo on a Background of Aspirin—Thrombolysis In Myocardial Infarction
FDA	Food and Drug Administration
